# Targeting PDE10A GAF Domain with Small Molecules: A Way for Allosteric Modulation with Anti-Inflammatory Effects

**DOI:** 10.3390/molecules22091472

**Published:** 2017-09-04

**Authors:** Ana M. García, José Brea, Alejandro González-García, Concepción Pérez, María Isabel Cadavid, María Isabel Loza, Ana Martinez, Carmen Gil

**Affiliations:** 1 Centro de Investigaciones Biológicas (CSIC), Ramiro de Maeztu 9, 28040 Madrid, Spain; anamariagarcia.1988@gmail.com (A.M.G.); ana.martinez@csic.es (A.M.); 2 Instituto de Farmacia Industrial, Facultad de Farmacia, Universidad de Santiago de Compostela, Campus Universitario Sur s/n, 15782 Santiago de Compostela, Spain; pepo.brea@usc.es (J.B.); alejandro.gonzalez@usc.es (A.G.-G.); mariaisabel.cadavid@usc.es (M.I.C.); mabel.loza@usc.es (M.I.L.); 3 Instituto de Química Médica (CSIC), Juan de la Cierva 3, 28006 Madrid, Spain; conchi@iqm.csic.es

**Keywords:** PDE10 inhibitors, allosteric modulators, cAMP, GAF domain, anti-inflammatory

## Abstract

Phosphodiesterase (PDE) enzymes regulate the levels of cyclic nucleotides, cAMP, and/or cGMP, being attractive therapeutic targets. In order to modulate PDE activity in a selective way, we focused our efforts on the search of allosteric modulators. Based on the crystal structure of the PDE10A GAF-B domain, a virtual screening study allowed the discovery of new hits that were also tested experimentally, showing inhibitory activities in the micromolar range. Moreover, these new PDE10A inhibitors were able to decrease the nitrite production in LPS-stimulated cells, thus demonstrating their potential as anti-inflammatory agents.

## 1. Introduction

Phosphodiesterases are the enzymes responsible for breaking down the cyclic nucleotides cGMP and/or cAMP, which are two of the most important signaling molecules in cells [1]. Presently, eleven families of PDEs are known in mammalians, being integrated by a total of 21 isoforms. They could be classified depending on their cyclic nucleotide specificity: PDEs 4, 7, and 8 are cAMP-specific while PDEs 5, 6, and 9 are cGMP-specific ones. By their side, PDEs 1, 2, 3, 10, and 11 can hydrolyze both types of cyclic nucleotides [2].

The wide variety of tissue expression in humans for the different families of phosphodiesterases makes them attractive targets for treating a vast number of disease states [3]. PDEs are metaloproteins that have two metal ions, magnesium and zinc, involved in catalytic activity. Structurally, they are composed by a highly conserved catalytic domain near the *C*-terminal, which contains a cyclic nucleotide binding site, as well as by a regulatory one, closed to the *N*-terminal, which shows more variation among the different PDEs families. Until very recently, the work in relation to these therapeutic targets was focused on blocking the cAMP and/or cGMP binding sites on the catalytic domain. Nevertheless, the high level of conservation among the 11 PDE families commonly leads to selectivity issues [4]. This fact, together with the often wide distribution of these enzymes and the presence of multiple splice variants for everyone, makes the search for specific PDE inhibitors a current challenge. For this reason, new approaches are needed in order to modulate PDE activity, avoiding problems of selectivity. One strategy is to focus on a PDE family with limited isoforms and/or limited tissue distribution, while another feasible approach consists of looking for allosteric modulators targeting specific regulatory domains of PDEs. This last approach is expected to modulate one specific PDE activity without affecting the others. Within the regulatory domain of these enzymes, the one named GAF (cGMP-activated PDEs, Adenylyl cyclase, and Fh1A) is found in PDEs 2, 5, 6, 10, and 11. This domain is characterized by containing a conserved aminoacid sequence NKFDE [5], which has been demonstrated to be necessary for the binding of cyclic nucleotides in PDEs and, consequently, for catalytic activity regulation [6,7]. Every PDE possesses two GAF domains, A and B, one of them being crucial for cyclic nucleotide binding, whereas the other one is found to be simply a structural requirement for these enzymes regulation [7]. Therefore, PDE activity regulation by targeting the GAF domain emerges as a promising approach for getting a better rate of selectivity.

Cyclic nucleotides have been found to be the endogenous ligand for PDEs GAF domains. In fact, while PDEs 2, 5, 6, and 11 specifically bind cGMP, the PDE10A GAF-B domain is the only one modulated by cAMP [5,7]. However, their sequence conservation and similarities in the cyclic nucleotide pocket architecture are lower in the GAF domains than in the catalytic ones, suggesting that GAF domain-binding PDE drugs may have higher PDE-selectivity and fewer side-effects than catalytic-site inhibitors.

Regarding their structure, PDE2A is the only PDE for which the X-ray structure of a near full-length has been reported, including both catalytic and regulatory domains [8]. However, other crystal structures for GAF domains of PDEs are available, bounded to the corresponding cyclic nucleotide. In fact, the GAF-A and B domains structure for PDE2A in complex with cGMP are available [6], as well as the structure of the PDE10A GAF-B domain bounded to cAMP [9]. Altogether, the different structures have provided the clue to propose a possible mechanism for the regulation of PDEs via GAF domain [8]. Presently, only two molecules able to modulate PDE5 activity through the binding to its GAF domain have been identified by high throughput screening using a chimera construction, which are being further characterized [10].

Furthermore, an important point to consider is the possibility to use these crystal structures of the GAF domains as a tool for drug discovery using a target-based strategy. With the aim of overcoming off-target difficulties of the competitive inhibitors bound to the catalytic domain of these enzymes, here we proposed a structure-based virtual screening for the identification of novel candidates able to target the regulatory domain of PDE10A using its reported crystal structure [9]. PDE10A is an important target for different diseases. Through its implication in cGMP signaling, PDE10A has been recently reported as a target for colorectal cancer [11]. However, it is often more known as a target of neurological disorders such as schizophrenia [12], Huntington’s disease [13], and Parkinson’s disease [14], and some of its inhibitors are in different phases of clinical development [15] due to its ability for cAMP modulation. The PDE10 inhibitors will be able to increase cAMP levels significantly to show neuroprotective properties [16]. The key role of cAMP in the anti-inflammatory response may be one of the biological bases for the therapeutic effects of PDE10 inhibitors because elevation in cAMP levels significantly show immunosuppressive and anti-inflammatory properties [17,18]. The final aim of this work is to discover allosteric modulators of PDE10A as new anti-inflammatory agents useful in different neurological diseases. 

## 2. Results and Discussion

### 2.1. Structure-Based Virtual Screening and Ligand Identification

In order to discover novel selective PDE10 inhibitors based on the GAF-B domain structure of human PDE10A (PDB: 2ZMF) [9], a virtual screening was carried out using our in house-chemical library. More than 800 heterocyclic molecules with desired CNS drug-like properties compose this collection called the MBC library [19]. The program used was Glide vs. 9.2 Schrödinger, centering the grid in the binding site of the cyclic nucleotide. To establish a valid virtual docking method, we used cAMP and cGMP as controls. 

**Figure 1:**
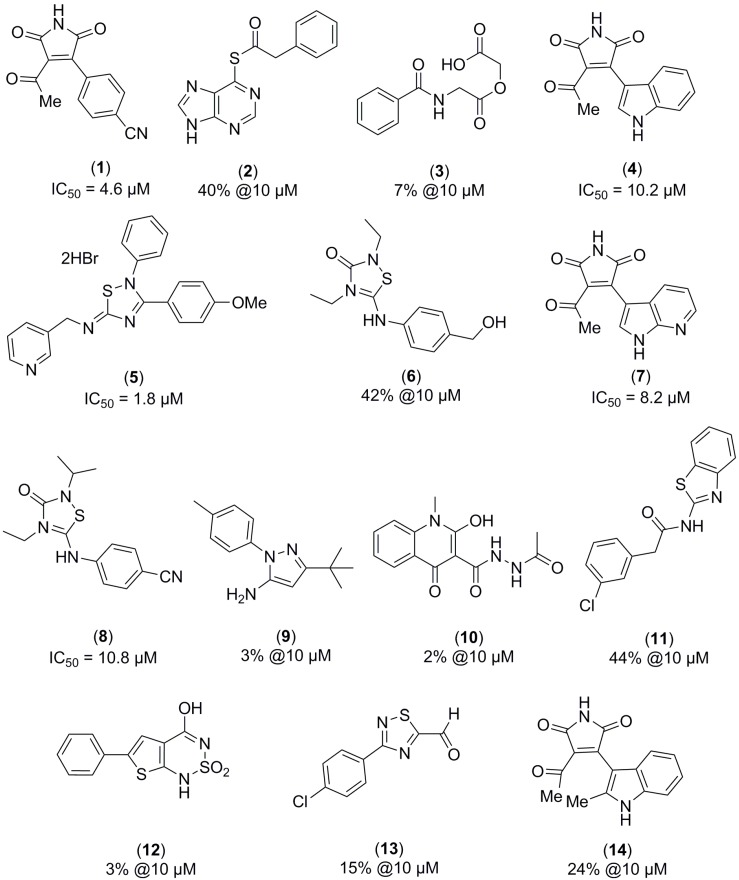
Molecules identified by virtual screening based on the GAF domain of PDE10A and its experimental enzymatic activity. Values of PDE10A inhibition are expressed as the percentage of inhibition at a fixed compound concentration of 10 µM or as the value of IC_50_. (Reference compound Papaverine: IC_50_ (PDE10A) = 0.019 µM [21]).

Once the virtual screening was carried out following workflows, screening, and post-processing analysis, as detailed in the Material and Methods Section, ligands with the best scoring functions were selected for visual inspection using Pymol^®^ (Schrödinger, Inc., New York, NY, USA). As a result, several molecules belonging to different heterocyclic families were identified (Figure 1). These compounds were tested against recombinant human full-length isoenzyme PDE10A following the procedure detailed in the Material and Methods Section. After the enzymatic evaluation of the hits virtually identified, two different chemical scaffolds emerged as potential new PDE10A inhibitor families: thiadiazole (compounds **5, 6,** and **8**) and maleimide (compounds **1**, **4,** and **7**). The thiadiazole family was reported previously as PDE7 inhibitors [20]; thus, we decided to focus our efforts on the maleimide family to further study their potential as PDE10A allosteric regulators.

**Figure 2:**
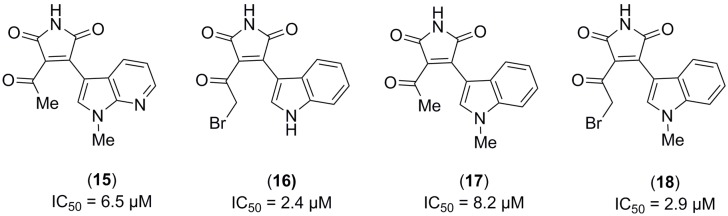
PDE10A inhibition of compounds (**15**–**18**), together with reference compound Papaverine. (Reference compound Papaverine: IC_50_ (PDE10A) = 0.019 µM [21]).

First of all, to check how the different substituents attached to the maleimide core may influence the biological activity, we evaluated a focused in-house small chemical library composed by maleimide related-molecules against the recombinant human PDE10A enzyme. The four maleimide derivatives tested were identified as PDE10A inhibitors in the micromolar range (Figure 2). 

Furthermore, to have a better insight into the theoretical binding mode of these new identified chemotypes, a comparative docking study was performed. The main goal of this in silico analysis was to determine the allosteric modulation behavior of the maleimides when they inhibit PDE10. We used the reported crystal structures of the catalytic domain of PDE10A (PDB code: 2OUP) and the GAF domain one (PDB code: 2ZMF), together with two PDE10A inhibitors: one targeting the catalytic site, the 4,5-bis(4-bromophenyl)-2-(2-chlorophenyl)-1*H*-imidazole (**19**) [22], and other able to bind to the GAF-B domain of the enzyme, the maleimide **1**. As controls to validate our docking protocol, we used both cAMP and cGMP. The stability of the binding mode for every ligand in the protein was evaluated using the scoring function given by Glide XP for every complex ligand-protein (Table 1).

The obtained results showed, firstly, that cAMP affinity for the catalytic binding site is higher than the cGMP one, according to the kinetic constant determined for every nucleotide [23]. Notably, in the case of GAF domain binding, the huge difference in affinity showed by both nucleotides can be highlighted, which agrees with the fact that cAMP tightly binds to the nucleotide cyclic pocket of the GAF-B domain [7], having almost no affinity for cGMP.

**Figure 3:**
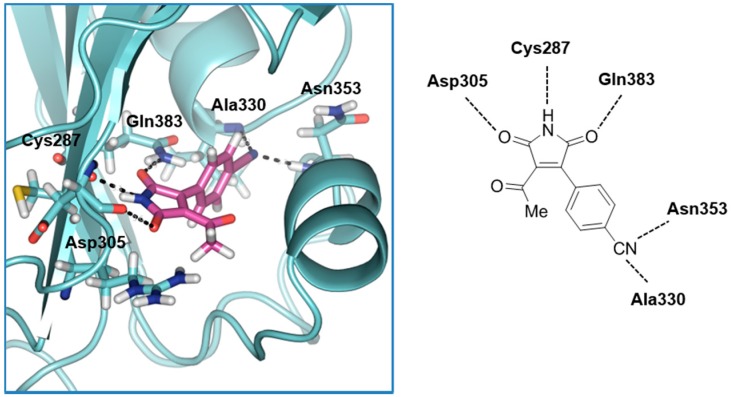
Binding mode of derivative **1** in the cAMP binding site of GAF-B domain of PDE10A (IC_50_ = 4.6 µM).

Similarly, the score functions for the PDE10 inhibitor previously described, imidazole **19**, show clearly its preference for the catalytic site regarding the GAF domain. Furthermore, in relation to ligand **1**, its scoring function is notably more stable for the GAF-B domain than the catalytic one. A deeper insight into the binding mode of compound **1** in complex with the GAF-B domain showed some stabilizing interactions which can explain the biological activity experimentally observed for this family of compounds (Figure 3). First of all, the NH group of the maleimide scaffold makes a hydrogen bond interaction with the carbonyl group of Cys287, an amino acid that has been reported to be important for cAMP binding. Moreover, the two carbonyl groups of the maleimide core interact with the amino group of Gln383 and the hydroxyl group of Asp305, respectively (not to mention that a π-cation interaction between the aromatic ring of maleimide and the amino group of Gln383 is found to be important, since this kind of aromatic substitution is found in every maleimide derivative). In addition to these conserved interactions among all maleimides, derivative **1** presents a double interaction of its nitrile group with Asn353 and Ala330.

### 2.2. Inhibition of Nitrite Production in LPS-Stimulated Murine Macrophages

**Figure 4:**
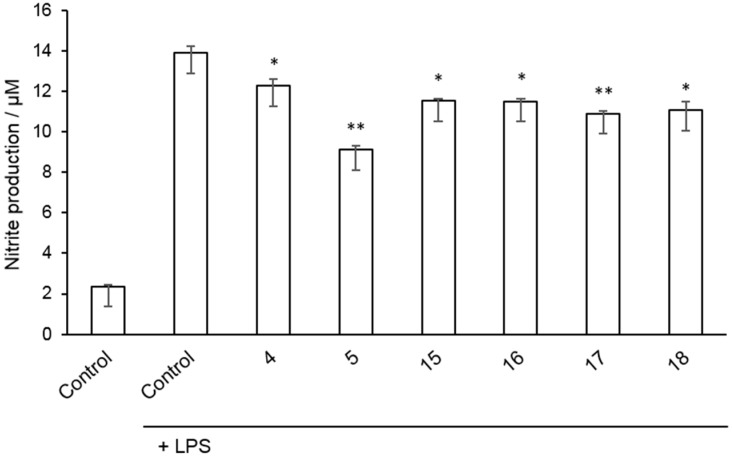
Raw 264.7 cells were incubated for 24 h with lipopolysaccharide (LPS; 10 μg mL^−1^) in the absence or presence of various Phosphodiesterase (PDE) inhibitors (10 μM), and the production of nitrite was evaluated by the Griess reaction. Cells were pretreated with inhibitors for 1 h before lipopolysaccharide (LPS) stimulation. Values represent the mean ± SD from two independent experiments. *: *p* < 0.05; **: *p* < 0.01 versus LPS-treated cells.

Finally, to explore the anti-inflammatory potential of the new allosteric PDE10A inhibitors here described, a cell-based assay was used. We have determined differences in nitrite production of Raw 264.7 cells after they were damaged with bacterial lipopolysaccharide (LPS) and treated with selected inhibitors. First, the effect of the compounds on cell viability was examined at various concentrations showing any significant cytotoxic effect in the present experiments (data not shown). Afterwards, the PDE10A inhibitors tested (**4**, **5**, **15**–**18**) significantly decreased nitrite production measured by Griess reaction (Figure 4). These results show the ability of allosteric PDE10A modulators to decrease inflammation in vitro.

## 3. Materials and Methods 

### 3.1. Computational Chemistry

#### 3.1.1. Protein and Ligand Preparation

To carry out the docking studies, the PDE10A enzymes employed were based on the crystal structure reported in the Protein Data Bank for the catalytic and the GAF-B domain (PDB code: 2OUP and 2ZMF, respectively). cAMP and cGMP were docked as reference compounds, which are the endogenous ligand of PDE10A. Protein was prepared for docking using Protein Prepared Wizard (Schrödinger, Inc., New York, NY, USA). Water molecules coordinated with both magnesium and zinc atoms were conserved for the docking development. Hydrogens were incorporated at neutral pH, and charges were added by the MMFF94 force field. Afterwards, the protein structure was minimized using that force field (gradient 0.01 kcal/mol).

Ligands were prepared using Ligprep (Schrödinger Inc.). Ligands MDL SDFile format (.sdf) were the input file, which were generated from structures in ChemDraw^®^ v12.0 (Waltham, MA, USA). The parameters for the generation of ligands were: the maximum number of atoms per ligand: 300; the maximum number of isomers per ligand: 8; the maximum number of tautomers per ligand: 6; the generation of the possible states at target pH 4–9. Afterwards, hydrogens were added by MMFF94 force field and the minimization of the generated structures was carried out using the same force field.

#### 3.1.2. Virtual Screening Workflow

The PDE10A GAF-B domain crystal structure (PDB code: 2ZMF) was used as a receptor and prepared as it has been previously indicated. The program used for the virtual screening procedure was Glide vs. 9.2 Schrödinger using the standard precision (SP) mode and flexible docking with ring sampling for the docking setup, with a maximum of 20 poses per ligand. Glide was used to generate a grid centered on the binding site of cyclic nucleotides. The box size was set to the value of 20 × 20 × 20 × 20 Å^3^. This box size encompasses the entire PDE10A binding pocket both in width and depth. The output solutions were ordered according to the scoring function assigned by the Glide program, selecting the ligands with the highest score to be then evaluated by visual inspection using Pymol v1.3 (Schrödinger, Inc., New York, NY, USA). After docking, the receptor-ligand complexes for the best poses were minimized with the MMFF94 force field (gradient 0.01 kcal/mol) using SybylX 2.0 (Certara, Inc., Princenton, NJ, USA).

#### 3.1.3. Comparative Analysis of Binding Modes Using Catalytic and GAF-B PDE10A Domains

The crystal structures of the PDE10A catalytic domain (PDB code: 2OUP) and the GAF-B ones (PDB code: 2ZMF) were used as a receptor. As reference ligands, cAMP and cGMP were employed. The extra precision (XP) mode of Glide vs. 9.2 Schrödinger was used as docking program, with a maximum of 20 poses per ligand. Glide was used to generate a grid centered on the binding site of cyclic nucleotides. The box size was set to the value of 20 × 20 × 20 × 20 Å^3^. The output solutions were ordered according to the scoring function assigned by the Glide program to be then evaluated by visual inspection using Pymol v1.3. These energies are shown in Table 1. After docking, the receptor-ligand complexes for the best poses were minimized with the MMFF94 force field (gradient 0.01 kcal/mol) using SybylX 2.0.

### 3.2. Radiometric Phosphodiesterase Inhibition Assay

The methodology used for measuring human recombinant PDE10A2 activity was based in a Scintillation Proximity Assay (SPA) from Perkin Elmer (TRKQ7090). The activity of the phosphodiesterase is measured by co-incubating the enzyme with [^3^H]cAMP and the hydrolysis of the nucleotide is quantified by radioactivity measurement after binding of [^3^H]AMP to the scintillation binding bead.

0.02 units of PDE10A (Calbiochem #524739) were incubated in a 96-well flexiplate with 0.05 μCi of [^3^H]cAMP and inhibitors in 100 μL of assay buffer (contained in the kit) for 20 min at 30 °C. After the incubation time, 50 µL of a solution of SPA-beads (approximately 1 mg per well) were added to each well and the plate was shaken for 1 h at room temperature. Finally, beads were settled for 30 min and radioactivity was detected in a Microbeta Trilux reader (Perkin Elmer, Shelton, CT, USA).

IC_50_ values were calculated by non-linear regression fitting using the GraphPad Prism. Data (radioactivity vs. log concentration) was fitted to a sigmoidal dose-response equation: Y = Bottom + (Top-Bottom)/(1 + 10^(logIC50-X) × n^), where Bottom and Top were the minimum and maximal inhibition for PDE, respectively, IC_50_ was the concentration of the compound that inhibited the PDE activity in a 50%, and n was the slope of the concentration-response curve.

### 3.3. Cell Viability Assays

Cell viability was measured using 3-(4,5-dimethylthiazol-2-yl)-2,5-diphenyltetrazolium bromide (MTT) assay. Briefly, the MTT solution (5 g/L) was added into each well and incubated at 37 °C for 4 h. After the removal of culture medium, 100 μL dimethyl sulfoxide was added into each well to dissolve the formazan crystals, formed by mitochondrial reduction of MTT. The optical density was measured at 532 nm using a microplate reader. The absorbance of the control group was considered as 100% of the cell viability.

### 3.4. Nitrite Quantification

The content of nitrite, one of the end products of NO oxidation, was monitored by a procedure based on the diazotidation of nitrite by sulfanilic acid (Griess reaction). Twenty-four hours after the incubation of Raw 264.7 cells with 0.4 µg/mL of LPS, 50 µL of sample aliquots were mixed with 50 µL of Griess reagent in 96-well plates and incubated at room temperature for 15 min. The absorbance (520 nm) of the mixture was measured on a microplate reader. The concentration of nitrite was calculated with the linear equation derived from the standard curve generated by known concentrations of sodium nitrite.

## 4. Conclusions

The allosteric regulation of enzymes as PDEs is an interesting approach for modifying enzyme activity avoiding off-target effects. Here, the search for allosteric modulators using a target-based approach focusing on the PDE10A regulatory GAF domain has allowed the identification of new hits that inhibit the enzyme in the micromolar range. In addition, these new PDE10A modulators have shown an ability to decrease inflammation in vitro by inhibiting LPS-induced nitrite production in murine macrophages. This effect may be due to the increase in the cAMP levels caused by the inhibition of PDE10A. These results open new horizons towards the modulation of PDEs in a selective way as an alternative for future treatments for inflammation-related diseases including neurological disorders.

## Figures and Tables

**Table 1:** Scoring functions for the binding of selected ligand to the catalytic and GAF domain of PDE10A (2OUP and 2ZMF crystal structures, respectively).

	Scoring Function			
Protein structure	cAMP	cGMP	Compound **19**	Compound **1**
Catalytic domain (2OUP)	−4.133	−2.816	−9.386	−4.803
GAF domain (2ZMF)	−8.071	−1.040	−1.395	−8.898
